# Acoustics Reveals the Presence of a Macrozooplankton Biocline in the Bay of Biscay in Response to Hydrological Conditions and Predator-Prey Relationships

**DOI:** 10.1371/journal.pone.0088054

**Published:** 2014-02-04

**Authors:** Ainhoa Lezama-Ochoa, Xabier Irigoien, Alexis Chaigneau, Zaida Quiroz, Anne Lebourges-Dhaussy, Arnaud Bertrand

**Affiliations:** 1 AZTI-Tecnalia, Marine Research Unit, Pasaia, Basque Country, Spain; 2 Institut de Recherche pour le Développement (IRD), UMR212 EME IFREMER/IRD/UM2, Sète, France; 3 IRD, LEGOS, UMR5566 CNES/CNRS/IRD/UPS, Toulouse, France; 4 Instituto del Mar del Perú (IMARPE), Callao, Peru; 5 IRD, UMR LEMAR CNRS/IRD/UBO, Plouzané, France; Dauphin Island Sea Lab, United States of America

## Abstract

Bifrequency acoustic data, hydrological measurements and satellite data were used to study the vertical distribution of macrozooplankton in the Bay of Biscay in relation to the hydrological conditions and fish distribution during spring 2009. The most noticeable result was the observation of a ‘biocline’ during the day i.e., the interface where zooplankton biomass changes more rapidly with depth than it does in the layers above or below. The biocline separated the surface layer, almost devoid of macrozooplankton, from the macrozooplankton-rich deeper layers. It is a specific vertical feature which ties in with the classic diel vertical migration pattern. Spatiotemporal correlations between macrozooplankton and environmental variables (photic depth, thermohaline vertical structure, stratification index and chlorophyll-a) indicate that no single factor explains the macrozooplankton vertical distribution. Rather a set of factors, the respective influence of which varies from region to region depending on the habitat characteristics and the progress of the spring stratification, jointly influence the distribution. In this context, the macrozooplankton biocline is potentially a biophysical response to the search for a particular depth range where light attenuation, thermohaline vertical structure and stratification conditions together provide a suitable alternative to the need for expending energy in reaching deeper water without the risk of being eaten.

## Introduction

Zooplankton play a key role in marine food webs [1] and their dynamics are closely related to the physical environment [2], [3]. As such, consideration of the factors that affect the distribution and abundance of zooplankton and its role in the ecosystem is key to understanding the impact of the environment on ecosystem functioning. Zooplankton distribution varies both horizontally and vertically across a continuum of spatiotemporal scales [4]–[6], but the factors that impact on the vertical patterns are usually different from those that influence the horizontal distribution [7]. Insight into the vertical distribution patterns of zooplankton is fundamental for understanding the dynamics and structure of zooplankton communities and their impacts on food web dynamics, global biogeochemical cycles, the effects of climatic change, and the potential yield of fisheries [8], [9]. Zooplankton exhibit relatively little active directed horizontal movement (beyond a few metres) but are capable of moving tens (mesozooplankton) to hundreds (macrozooplankton) of metres vertically in reaction to physical and chemical gradients, diel changes in light level, predation and food resources [10]–[13]. Furthermore, interactions between vertical current shear or random turbulence and vertical migration allow zooplankton to forage in widely-separated areas with little energy expenditure. This, however, results in greater horizontal spreading of macrozooplankton patches over time compared to less-migratory mesozooplankton [14], [15]. It is therefore very important to determine the proximate environmental factors that govern the vertical distribution patterns of macrozooplankton, beyond the widespread but basic documentation describing the diel vertical migratory behaviour of mesozooplankton.

One of the main limitations for understanding the processes that determine the distribution of zooplankton is the low spatial and temporal resolution of the net tows data [6]. However, in recent years, the advancement of acoustic methods have made it possible to observe a large number of communities, including zooplankton communities, at a large range of horizontal scales ranging from a few meters to that of a complete survey of hundreds to thousands of km (e.g.[16]–[18]). Acoustic data have revealed small-scale features in zooplankton distributions that have been, at best, under-sampled, but in most cases completely overlooked.

Studies concerning zooplankton in the Bay of Biscay, have until recently, focussed on trying to understand how climate affects the distribution of zooplankton [19]–[21], while most of the information related to the species composition and abundance of zooplankton has been directed at the micro- and meso-zooplankton components [22]. Information regarding other important components such as macro- or gelatinous zooplankton (>∼2 mm in size) is scarce given the difficulty in effectively capturing and thus quantitatively sampling these larger organisms with the use of plankton nets [22]. Macrozooplankton react to both visual and mechanical disturbances and are therefore known to avoid net sampling, particularly when commonly-used vertical tows are conducted [23], [24]. Consequently, although oceanic, coastal-neritic, and estuarine mesozooplankton communities have been studied extensively, these findings are not really representative of the macrozooplankton component. Besides, most of these studies address the horizontal mesoscale variations in distribution of zooplankton with little information pertaining to their vertical distribution.

A recent study [25], conducted in the Bay of Biscay, used acoustic data to describe the horizontal distribution of the macrozooplankton component and its scale-dependent relationships with pelagic fish. To further this work, we focus here on the vertical dimension and examine how environmental conditions influence the vertical distribution patterns of macrozooplankton at the onset of spring water-column stratification. In particular, we aim to quantify the relative roles of abiotic and biotic (predator-prey relationships) features in influencing the macrozooplankton vertical distribution during day and night periods. The vertical distribution of organisms stems from a compromise between eating and not being eaten, which manifests in growth and mortality. Thus, by taking into account both the temporal (diel period and survey duration) and spatial scales (geographical areas and ecological domains), we aim to explain the vertical distribution patterns exhibited by macrozooplankton with consideration of the following environmental parameters: vertical thermohaline structure (temperature, salinity and density) and associated stratification, primary production (chlorophyll-a concentration), photic depth (daytime period), and predator vertical distribution (fish biomass estimated acoustically).

## Materials and Methods

### Acoustic Data Acquisition

Acoustic data were recorded with a Simrad EY60 split-beam scientific echosounder operating at 38 and 120 kHz (Kongsberg Simrad AS) during a routine scientific survey performed in spring (April–May) 2009 in the Bay of Biscay as part of the BIOMAN program (AZTI project) (see [25]). BIOMAN surveys estimate the spawning biomass of anchovy *Engraulis encrasicolus* from the daily egg production method. These multi-disciplinary surveys also collect acoustic data as well as a large number of mesozooplankton (0.2 to 2 mm in size) samples, information on hydrographic parameters (see below), and pelagic fish sampling by means of pelagic trawl hauls [26].

**Figure 1:**
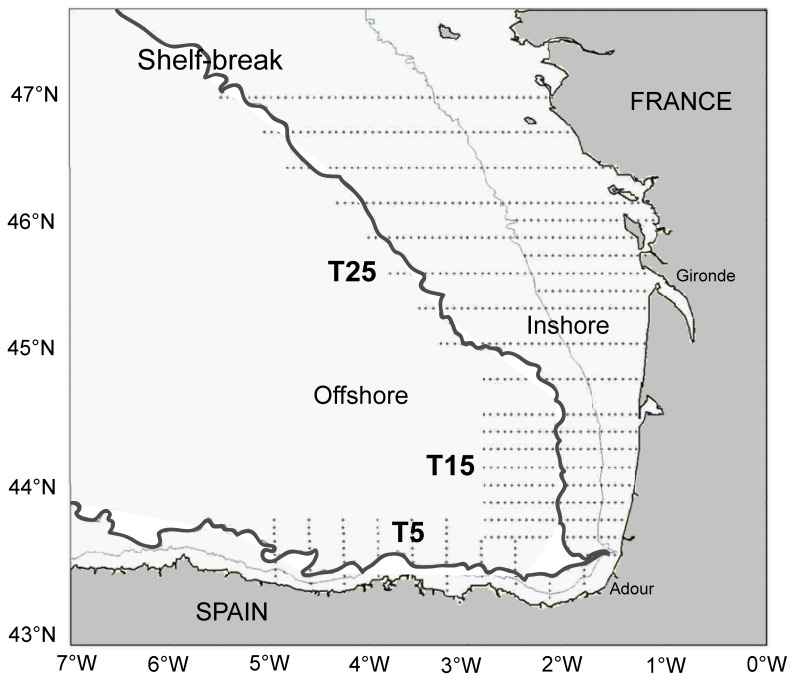
Study area. River mouths, shelf areas (coasts) and ecological domains (inshore-offshore) are indicated. The dotted lines show the survey track. T1, T15 and T25 refer to transects presented in Figure 7.

The sampling area covered the Bay of Biscay (the Cantabrian Sea and off the French coast), with the western survey limit at 5°W (beginning of the survey) and the northern limit at 47°N (end of the survey) (Fig. 1). Sampling was carried out during both day and night and the survey design was a combination of systematic and adaptive schemes. The systematic scheme was based on cross-shelf transect lines running offshore from the coast (bottom depth ∼20 m) to beyond the shelf break. Transects were parallel, regularly spaced and perpendicular to the coast with an inter-transect distance of 15 nautical miles (nm). Standard transects occurred generally 6 to 10 nm off the shelf break when no anchovy eggs were found further from the shelf break. Otherwise, transects were prolonged as long as eggs were detected and then stopped when no eggs had been found within 6 nm. This adaptive scheme was adopted to ensure that the entire anchovy spawning area was sampled.

The echosounder, which was calibrated according to standard methods [27], sampled the water column down to depths of 300 and 500 m for the 120 and 38 kHz channels, respectively. For the purposes of this study, however, we only considered the water column in the depth range from 10 to 100 m. The upper depth limit was chosen to ensure that measurements were made within the far field of the transducers [28]. The bottom depth limit was chosen to eliminate electronic noise which occurred at depth >150 m in the echograms (the survey was performed onboard a commercial vessel) and to coincide with the maximum depth at which hydrographic data was collected. Acoustic data were selected, classified and analysed with Echoview® (Myriax) and MATLAB (MathWorks) software.

### Bi-frequency Classification Method

We categorized acoustic echoes using a bi-frequency acoustic method developed by [17]. This method uses the 38 and 120 kHz frequencies to extract continuous high-resolution information on the spatiotemporal patterns of pelagic fish and crustacean macrozooplankton [17], [25]. Apart from a few modifications, the original method, as used by [25], was applied virtually unchanged.

#### Pre-processing: removing noise and resampling

First, the ping number and position between echograms were synchronized using the matching ping time algorithm from Echoview. Then, the echograms were cleaned by defining and eliminating bottom echoes or regions containing parasite noise (unwanted signals present in the medium but independent of the echosounder transmission; [29]) or a ‘school tail’ (diffuse ragged tail below the more solid mark of the school).

Acoustic scattering is stochastic, and thus it is necessary to average acoustic measurements to reduce natural variations in the data [30]. Following the recommendations of [30], the bi-frequency echograms were resampled in common elementary cells with a length of 1 ping and a height of 0.80 m (from 4 raw cells 0.2 m in height). Finally, the noise associated with the acoustic absorption for both frequencies was eliminated [31], [32].

#### Discriminating acoustic scatterers

Zooplanktonic organisms comprised of weakly-scattering material and having acoustic properties similar to the medium in which they occur are usually called ‘fluid-like’ zooplankton [33]. The fluid-like group includes euphausiids, copepods, salps, siphonophores (without gas inclusion) and other large crustacean zooplankton (e.g. squilla larvae, munidae and other decapod larvae).

By combining the difference (ΔMVBS_120−38_) and sum (+MVBS_120+38_) of the mean volume backscattering strength (MVBS) between the frequencies (120 and 38 kHz), this method makes it possible to determine and quantify the crustacean macrozooplankton biomass. Therefore, based on observations (expert scrutinizing of the echograms) and exploratory analysis (distribution of volume scattering strength (*S*v) frequencies), a threshold value of −138 dB for the sum echogram (+MVBS120+38) was chosen and used as a Boolean mask (true for values above the threshold) to extract fish data (above −138 dB) from other scatters (below −138 dB) and create ‘fish’ and ‘no fish’ (still not free from weak fish scatters) echograms at each frequency (Fig. 2a in [25]).

**Figure 2:**
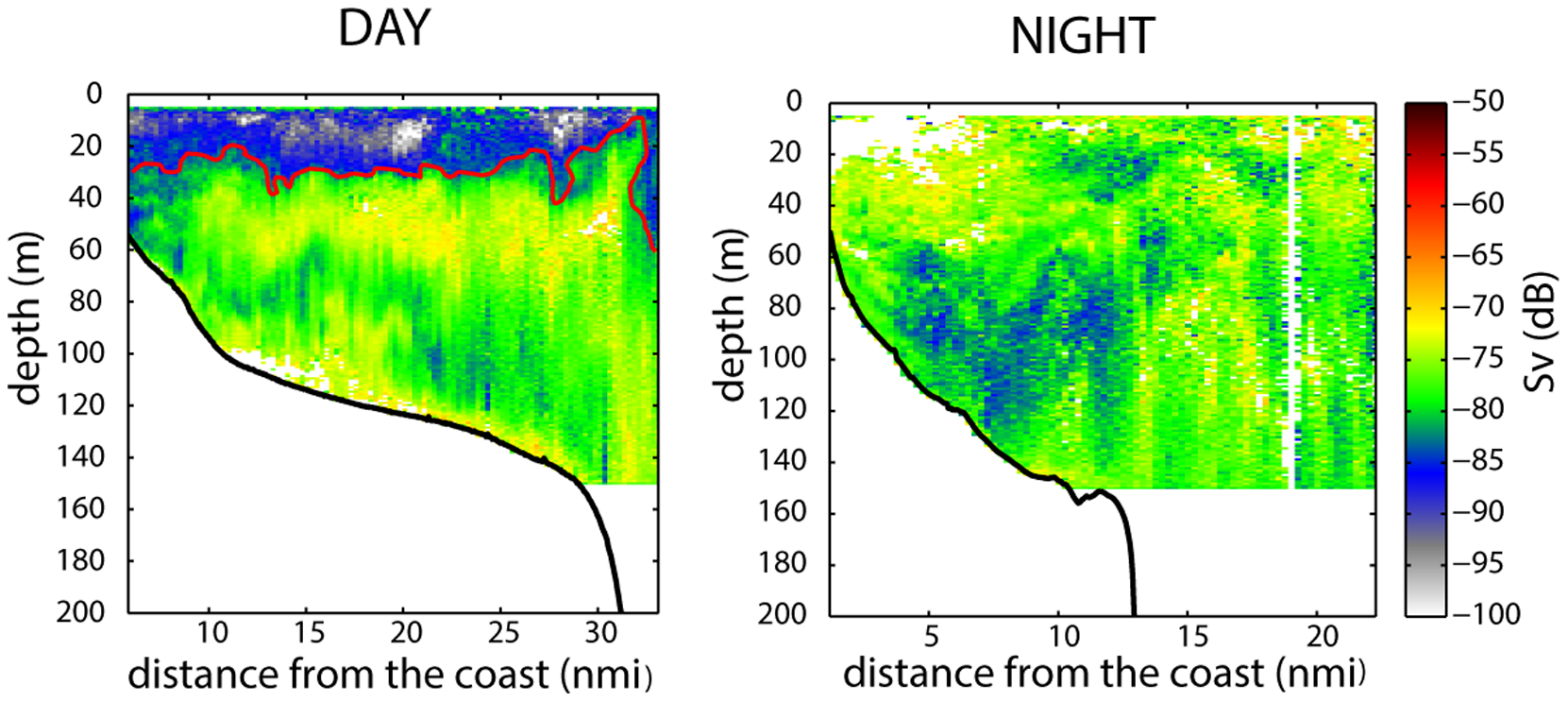
Fine scale representation of macrozooplankton diel vertical behaviour. Echograms of the macrozooplankton backscattering strength (*S*v in dB re. 1 m^−1^) show the differences in distribution between the two diel periods, which makes it possible to define a “biocline” (red solid line) as the depth where the cumulated sum of acoustic echoes (*S*v) from the macrozooplankton community reaches 5%.

With the exception of mackerel *Scomber scombrus*, most of the pelagic fish present in the Bay of Biscay, in particular anchovy (*Engraulis encrasicolus*), sardine (*Sardina pilchardus*), chub mackerel (*Scomber japonicus*) horse mackerel (*Trachurus trachurus*) and the mesopelagic fish *Maurolicus muelleri* and *Benthosema glaciale* have swimbladders. Therefore, any reference to ‘fish’ in this study is to swimbladder-bearing fish. Swimbladder-bearing fish have a slightly higher backscatter at 38 than 120 kHz [34], but there are a few cases of positive ΔMVBS_120−38_ (up to ∼+3 dB) in the fish data. We thus refined the data from the fish echograms by applying a second Boolean mask in order to keep only the targets for which ΔMVBS_120−38_< +3 dB. Although this constraint (∼+3 dB) also included mackerel in this group [32] we assumed that any reference to fish in this study pertains mainly to swimbladder-bearing fish. Given that the swimbladder is responsible for 90–95% of the backscattering strength of a fish [35] it is obvious that swimbladder fish would in any event strongly dominate the ‘fish’ acoustic biomass. Then, the fluid-like group was extracted from the ‘no fish’ echograms by applying a third Boolean mask to select the targets with a positive ΔMVBS_120−38_ greater than +3 dB. Targets with a negative ΔMVBS_120−38_ were classified as ‘others’ (‘blue noise’ in [17]). This last group included all targets other than fluid-like zooplankton and swimbladder-bearing fish (mainly fish larvae and gelatinous and gas-bearing siphonophores). Finally the classification groups were smoothed and mapped onto the original data, and maximum and minimum echointegration thresholds were applied to each class. More details of the methods applied in the Bay of Biscay can be found in [25].

#### Acoustic biomasses

As mentioned above, the fluid-like group mainly includes euphausiids, copepods, salps and siphonophores (without gas inclusion). In the Bay of Biscay, salps are not common on the shelf but can appear on the slope and farther offshore [36]. Likewise, siphonophores without gas inclusion have a very low biomass [37]. Therefore, as showed in [25], the fluid-like field extracted in this study was mainly composed of euphausiids, but also large copepods.

In the absence of a strict definition for the size range of macrozooplankton, we classified any zooplankter larger than 2 mm as macrozooplankton. This definition theoretically includes all the organisms that can be detected using the bifrequency method [38]. This study focused on the macrozooplankton community as a whole using the volume backscattering strength (Sv in dB ref 1 m^−1^) or the volume backscattering coefficient (s_v_ in m^−1^) as an index of its volumetric density.

The fish group corresponded to all small pelagic swimbladder-bearing fish, in particular the most abundant, anchovy, sardine and horse mackerel. Fish volume backscattering strength (Sv) was converted into an acoustic nautical area scattering coefficient (NASC in m^2^ nm^−2^), as an index of the fish biomass [39].

#### Defining diel periods

Diel vertical migration is a common behaviour for zooplankton and nekton. Its effects can be detected at almost all spatial scales (e.g. [6]). The diel vertical migration of macrozooplankton can affect acoustic density estimations because some species may migrate below the range of the acoustic sample (100 m in our study). Thus, in order to use consistent diel periods, we processed day and night acoustic data independently, and data from the twilight periods ±15 min were discarded.

#### Variables of interest

*Macrozooplankton and fish vertical distribution:* Besides the acoustic indices (Sv, s_v_, NASC), two spatial indices were used to describe the vertical patterns of macrozooplankton and fish: the displacement of the centre of gravity and the population inertia. In a two-dimensional plane, the centre of gravity represents the population’s mean location with a vector of two coordinates. The inertia, whose unit is a surface (typically nm^2^), quantifies the population’s spatial dispersion around its centre of gravity [40]. When sampling is regular, the following equations are used to calculate the centre of gravity (*CG*) and the inertia (*I*):
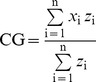


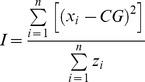
where *x* is the location of sample *i* (short for the usual two-dimension notation (*x, y*)) and *n* is the total number of samples; *z_i_* is the value of the sample at (*x_i_,y_i_)*.

The centre of gravity of macrozooplankton (CG_macro_) and fish (CG_fish_) aggregations were used as a proxy for characterising the vertical patterns of these organisms.

*Biocline:* A strong gradient in zooplankton biomass corresponding to the uppermost portion of the detected biological assemblage was observed during the day with densities increasing from the surface zone, which was almost void of zooplankton to deeper waters (Fig. 2). The interface, in which the zooplankton biomass changes more rapidly with depth than it does in the layers above or below, was termed the ‘biocline’. To determine the biocline depth the vertical gradient of zooplankton biomass was first calculated. Indeed, gradients are commonly used in a similar way to assess the thermocline, halocline or pycnocline depth. The distribution of zooplankton is, however, very patchy and the acoustic strength varies over several orders of magnitude. Hence, the estimation of the biocline depth would not be robust if only a single gradient threshold were to be used. Instead, the vertically cumulative sum (expressed as a percentage) of acoustic echoes (s_v_) originating from the macrozooplankton community, and integrated downward from the surface to a depth of 100 m or the bottom, was evaluated against several thresholds (Fig. 2). Different thresholds (where the threshold corresponds to the percentage of the echo over the entire range) in 1% increments between 1% and 10% and the resultant biocline patterns in different conditions (day-night, offshore-inshore) were visually inspected. A 5% threshold (the depth at which 5% of the total backscattering from the water column is reached) was found to be the best compromise during the day. Lower thresholds (<5%) tended to underestimate the biocline depth, whereas higher thresholds (10%) could potentially give rise to erratic macrozooplankton patterns (i.e. when a few strong scatterers were distributed below the main boundary). Thus, although possibly confusing, the biocline was here defined using a cumulated sum (and a visual check) instead of a vertical gradient.

At night, however, the macrozooplankton was distributed uniformly throughout the water column (0 to 100 m) and no biocline (i.e. abrupt change in biomass) could be observed. To estimate the depth of the biocline the data were processed over horizontal sampling distance units of 0.25 nm.

### Hydrological Data

Hydrographic stations were occupied every 3 nm along each cross-shelf transect. Conductivity, temperature and depth data loggers (CTD RBR XR420) were lowered to a maximum depth of either 100 m or 5 m above the bottom at shallower depths. Salinity and temperature profiles, initially acquired at 6 Hz, were vertically averaged at 1 dbar intervals. Seawater density (ρ) was estimated using the UNESCO equation of the state of seawater [41].

**Figure 3:**
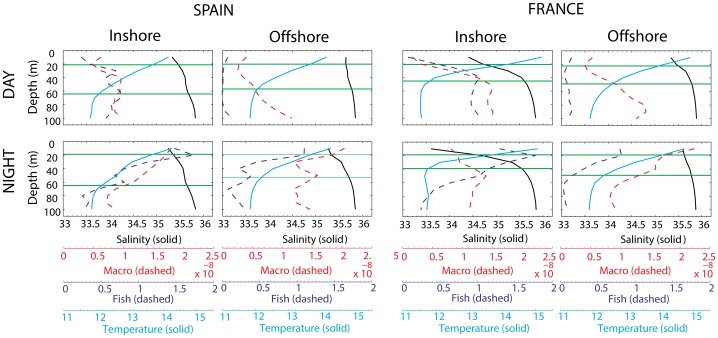
Overall day-night vertical profiles of physical and biological variables. Temperature (light blue solid line), salinity (black solid line), macrozooplankton biomass (red dashed line), and fish biomass (dark blue dashed line) vertical distributions are represented in inshore and offshore domains in the Spanish and French areas. The upper and lower thermocline are represented as green horizontal lines.

The thermocline and halocline, which separate the relatively warm and fresh surface waters from the cold and salty subsurface water (e.g. Fig. 3) in the Bay of Biscay, correspond to subsurface layers characterized by strong vertical temperature and salinity gradients. Thus for each of the acquired hydrographic profiles we used smoothed temperature and salinity gradient profiles and defined the thermocline and halocline as the layers in which gradient values exceeded a given threshold. The upper and lower thermocline and halocline correspond to the top and bottom of these layers. The thermocline and halocline depth was then defined as the depth at which the smoothed vertical temperature and salinity gradients reached their highest respective values.

We also used the Brunt-Väisälä frequency (*N* in s^−1^) as another descriptor of the water column. This buoyancy frequency, defined as 
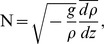
 where *g* is the gravitational acceleration, relates to the vertical density gradients and is an index of the water column stratification. For each vertical profile, the maximum value of *N* can be used as an indicator of the stratification strength. In general a sharper and thinner thermocline is associated with more intense stratification.

### Satellite Data

Daily satellite-derived information at 4×4 km^2^ spatial resolution from MODIS/Aqua was used to complement the *in situ* hydrographic dataset. The parameters considered were the diffuse attenuation coefficient (k490) and chlorophyll-a concentration that were spatiotemporally interpolated to coincide with the location of the hydrological stations. Attenuation, defined as the sum of scattering and absorption of light in seawater, is an indicator of the turbidity of the water column. A lower attenuation depth corresponds to reduced water clarity. Thus, this parameter can be used as a rough estimate of the depth at which 1% of the daylight penetrates the water (1/attenuation) – the depth that we considered as the photic depth (m).

### Biological Data

Anchovy and other small pelagic fish species, including sardine, mackerel (*Scomber scombrus* and *Scomber japonicus*), blue whiting (*Micromesistius poutassou*) and horse mackerel dominated the pelagic trawl catches during the survey [42]. The lack of biological sampling of some biotic and physical parameters (i.e. processed net samples of zooplankton were not available for this survey and vertical profiles of chlorophyll-a could not be obtained due to technical problems) resulted in a lack of accurate information on biological components other than fish. However results will be discussed based on previous references in the area.

### Defining Spatial and Temporal Effects

For each diel period (day/night), the macrozooplankton vertical distribution patterns and environmental variables were analysed in: (i) two geographical areas (Spanish and French areas) based on their different mesoscale oceanographic structures and hydrographical regimes [43]; and (ii) two ecological domains: the inshore region, from the coast to the shelf break (∼200 m depth); and the offshore region, from the shelf break (∼200 m depth) out to beyond a bottom depth of 1000 m (Fig. 1).

#### Regional scale

At a regional scale, for each diel period (day/night), the mean vertical profiles of macrozooplankton, fish, temperature (and upper/lower thermocline) and salinity were compared between the geographical areas and ecological domains.

#### Local scale

To quantify the relationships between CG_macro_ and the environmental parameters (temperature, salinity, stratification, photic depth, chlorophyll-a and CG_fish_), the horizontal resolution of the parameters was set to 1 nm. Student’s *t*-tests were used to determine whether significant diel differences in CG_macro_ and inertia between the inshore and offshore regions existed.

Correlation analyses (through the use of scatter plots) were applied to: (i) study the temporal distribution of CG_macro_, biocline and the environmental variables in relation to the diel periods, geographical areas and ecological domains; and (ii) study the relationships between the vertical patterns of macrozooplankton distribution (CG_macro_ and biocline) and the environmental variables.

As previously reported [25], these data are generally autocorrelated at a scale of 1 nm. The impact of the autocorrelation on the correlation coefficients was taking into account through further statistical testing as developed by [44], [45].

## Results

### Environmental Oceanscape

As in other temperate seas, oceanographic processes in the Bay of Biscay are greatly influenced by seasonal variability. In early spring, a rapid temperature increase is observed in the near-surface layers. The warming begins in the south-eastern part of the Bay, and progressively extends northward over the French shelf [46]. Since the Spanish area was sampled at the beginning of the survey in early May 2009 we observed an increase in stratification over time as our survey progressed from the Spanish to the French regions. Off Spain, the sea surface temperature was low and the stratification relatively weak. In contrast, by the time that the central French shelf and coastal area were sampled, thermohaline stratification had already set in. Stratification was highest in the inshore regions, especially in the vicinity of river mouths, where the strengthening of the seasonal thermocline was associated with a strong halocline brought about by river discharge (mainly Adour and Gironde, Fig. 1). Further north, the stratification process was probably still in progress and the level of stratification was moderate.

### Regional Scale

General patterns, dependent on the diel period and ecological domain considered, emerged upon inspection of the vertical profiles of hydrological conditions, macrozooplankton and fish (Fig. 3). Vertical gradients in temperature were stronger and the thermocline narrower and shallower over the shelf area compared to the offshore domain, particularly in the French region. Clear vertical salinity gradients were only observed in the French inshore region, where large river plumes generally occur and near-surface salinity decreases (Fig. 3). During the day, the macrozooplankton density increased with depth due to the diel vertical migration from the surface toward deeper layers. The mean vertical profile of macrozooplankton density indicated two maxima in the inshore regions but only one in the offshore regions. Shallower maxima ranged between 40 and 60 m depth, whereas the deeper maximum was observed at depths ranging from 80 to 100 m (and probably even deeper, offshore of our sampling limits). The vertical sampling range (100 m) precluded observations of the entire vertical extent of macrozooplankton distributions. Vertical profiles of fish biomass exhibited a similar pattern in the inshore regions, with two maxima which almost overlapped in depth with those of macrozooplankton. In contrast, mean fish abundance was much reduced in the offshore regions with no clear vertical pattern apparent. At night, however, macrozooplankton and fish were mainly distributed in the surface layers (0–40 m).

Generally, observations at the regional scale suggest that the vertical patterns of fish and macrozooplankton are very similar; with organisms ascending towards the surface at sunset and descending to deeper waters at sunrise. Furthermore, the thermocline appears to play an important role in the distribution of organisms with a higher biomass distributed below the thermocline during daytime and above it at night.

### Local Scale

#### Exploratory analysis

**Figure 4:**
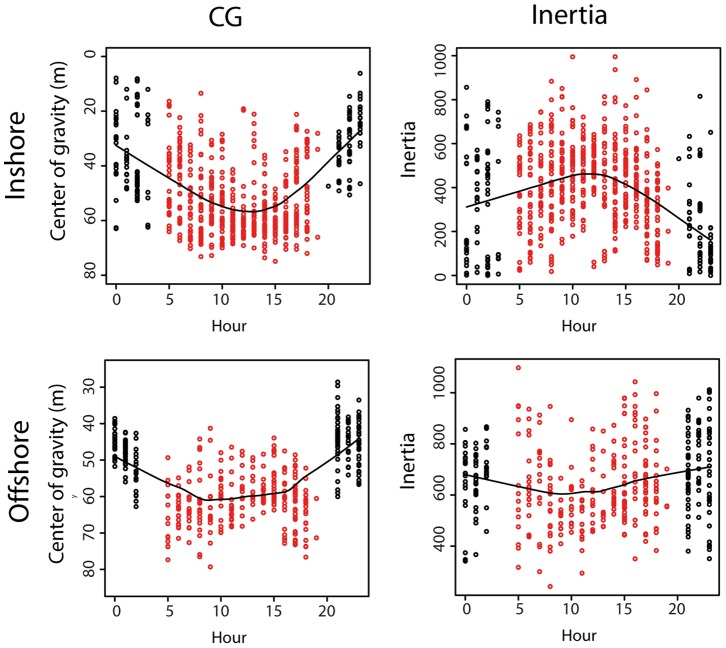
Day-night distributions of macrozooplankton. The centre of gravity (CG) of and the related inertia are analyzed according to the inshore and offshore regions. Day distributions are represented in red and night distributions in black. The black solid lines shows the smoothed distribution of the scattered data.

In accordance with the observation that macrozooplankton backscattering strength exhibited marked diel vertical migration behaviour (Fig. 2), the centres of gravity of the macrozooplankton distribution were also significantly deeper during the day than at night in both the inshore and offshore regions (Fig. 4, *t*-test *p*-value <0.001). Additionally, CG_macro_ were also slightly deeper at night in the offshore regions (Fig. 4) compared to the inshore regions whereas inertia increased significantly during the day in the inshore region (Fig. 4, *t*-test *p*-value <0.001) but decreased significantly (although to a lesser extent) in the offshore region (Fig. 4, *t*-test *p*-value <0.001).

#### Spatiotemporal analysis of biophysical factors

**Figure 5:**
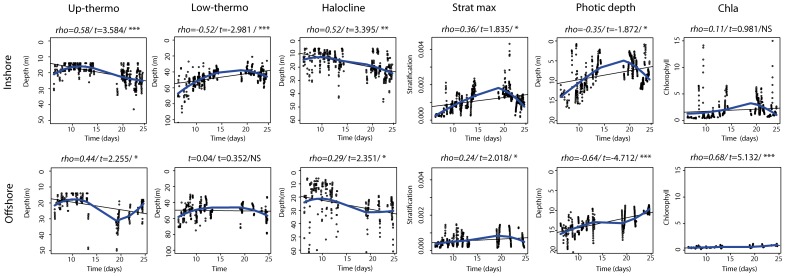
Temporal correlations of the physical variables. Analyses were done according to inshore and offshore ecological domains. Scatter plots include a linear fit (black solid line) and loess smoothing (blue solid line) to illustrate the sign of the correlation.

**Figure 6:**
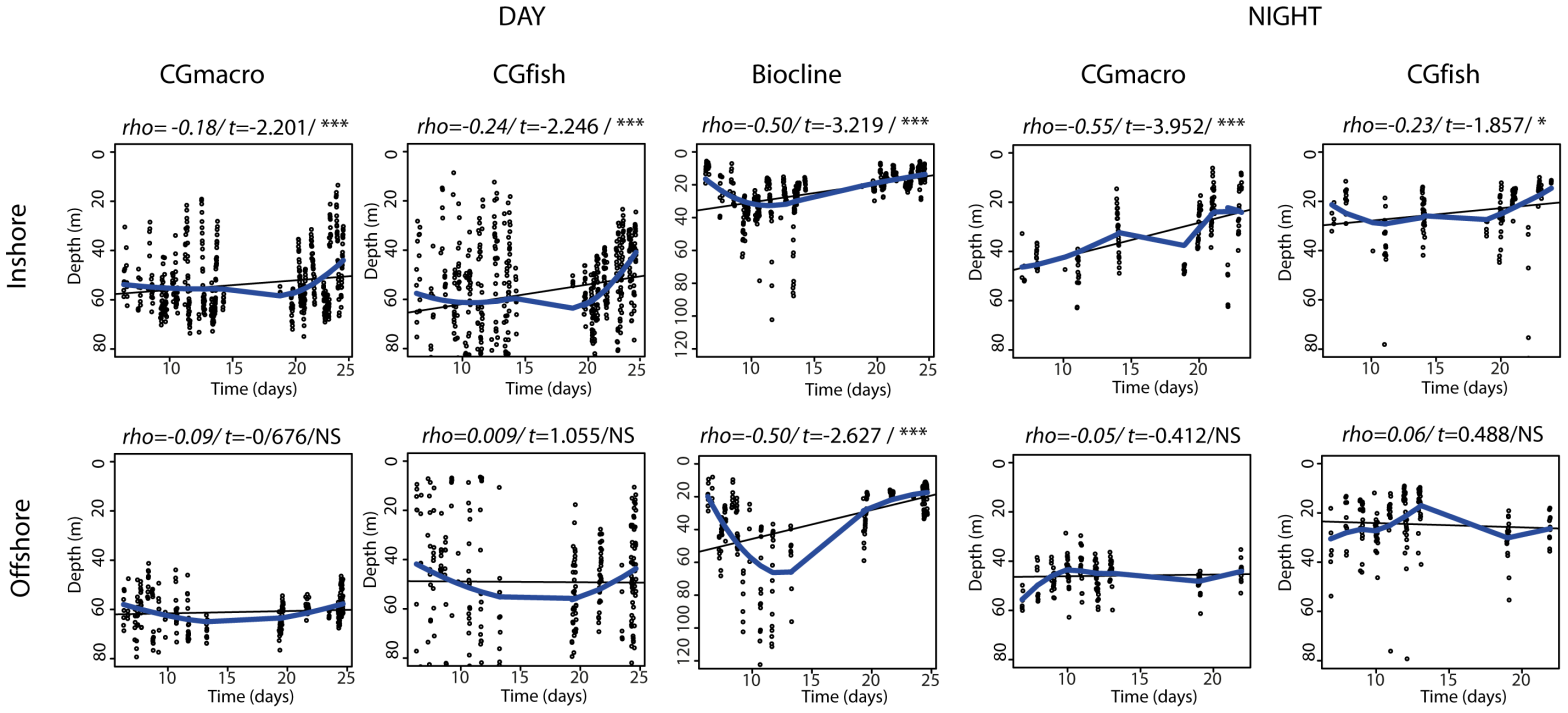
Day-night temporal correlations of macrozooplankton (CG_macro_ and biocline) and fish (CG_fish_). Analyses were done according to inshore and offshore ecological domains. Scatter plots include a linear fit (black solid line) and loess smoothing (blue solid line) to illustrate the sign of the correlation.

Oceanographic, macrozooplankton and fish vertical patterns showed clear temporal variation during the survey period and between regions (Figs. 5 and 6). These temporal variations also corresponded to spatial variations along the cruise track. In the inshore region a significant deepening of the upper thermocline and halocline was observed over time, whereas the lower limit of the thermocline layer became progressively shallower. The gradual narrowing (and deepening) of the thermocline layer resulted in increased stratification within the thermocline (Fig. 5). The photic depth decreased over time in the inshore region whereas the chlorophyll-a concentration exhibited no significant temporal trend. The chlorophyll-a distribution was, nonetheless characterised by three local peaks of high concentration coinciding with the Cap Breton and Cap Ferret canyons and the Gironde river plume.

In the offshore region, the upper limit of the thermocline and the halocline showed a similar deepening trend as was observed in the inshore regions, but here they occurred slightly deeper. Conversely, no significant trend was observed for the lower limit of the thermocline, which was located at ∼50 m depth in the offshore region (Fig. 5). The progressive deepening of the upper thermocline, coincident with a stable lower thermocline depth, gave rise to a slight increase in stratification. The photic depth was slightly deeper in the offshore region compared to closer inshore but a similar decreasing trend was observed. A significant increase in chlorophyll-a concentration over time was also observed along the offshore region.

The centres of gravity of macrozooplankton and fish deepened significantly with time in the inshore region during both diel periods (Fig. 6). In contrast, there were no significant temporal trends in the centres of gravity in the offshore region (Fig. 6).

**Figure 7:**
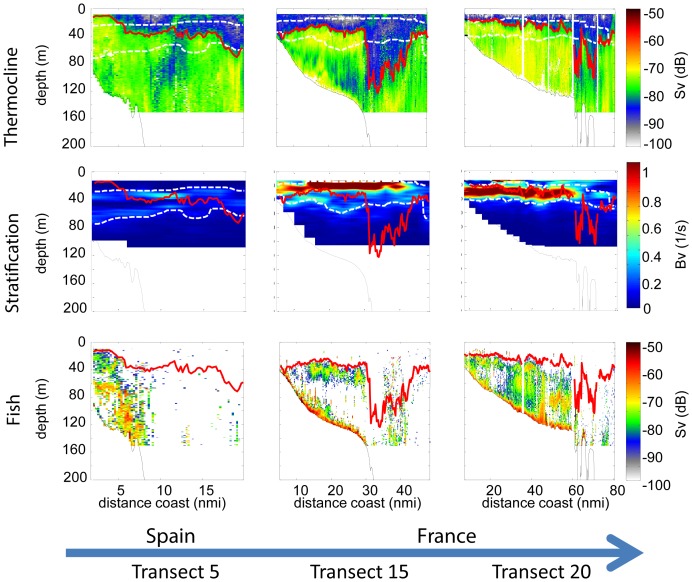
Along transect fine scale distribution of hydrological and biological parameters. Representation of backscattering strength (*S*v in dB re. 1 m^−1^), stratification and fish acoustic backscattering in relation to the biocline pattern (red solid line) during the survey period (transect 5, 15 and 20). The upper and lower limits of the thermocline layer are represented by white dotted line.

The biocline was located within the thermocline layer, except in the slope region, where it extended much deeper than the lower limit of the thermocline (Fig. 7). When stratification was intense and the pycnocline relatively deep (as along the cross-shore transect T20, located in the area of the Cap Ferret canyon, Fig. 7), the biocline depth coincided with the depth of maximum stratification. Under these conditions, the vertical distribution of fish was similar to that of the macrozooplankton with virtually no fish or macrozooplankton observed above the biocline (Fig. 7). The biocline got progressively shallower in both the inshore and offshore regions as the survey progressed (Fig. 6).

#### Macrozooplankton-environment interactions

Correlation analyses and scatter plots, performed to quantify the relationships between CG_macro_ and environmental variables (Figs. S1 and S2, Table 1) show that CG_macro_ and stratification index were negatively correlated during the day, in the offshore region of the Spanish area. Besides, CG_macro_ was negatively correlated with the stratification index in the inshore region of the French area, and positively correlated with photic depth in both the inshore and offshore regions. A positive correlation between CG_macro_ and CG_fish_ was noted in both the Spanish and French areas, irrespective of region (Figs. S1 and S2, Table 1).

**Table 1:** Correlations between CG_macro_ and the environmental variables according to the diel period, the area (Spain or France) and the ecological domains (inshore, offshore).

	CG_macro_									
	Day				Night					
	Spain		France		Spain				France	
	Inshore	Offshore	Inshore	Offshore	Inshore		Offshore		Inshore	Offshore
	(n = 25)	(n = 40)	(n = 263)	(n = 218)	(n = 21)		(n = 40)		(n = 138)	(n = 151)
Stratification	rho = −0.30	rho = −0.55	rho = −0.33	rho = −0.018	rho = 0.87	rho = −0.82		rho = −0.42		rho = −0.10
	t = −1.80/NS	t = −3.93/***	t = −4.12/***	t = −0.17/NS	t = 8.58/***	t = −6.52/***		t = −3.87/***		t = −0.59/NS
Photic depth	rho = 0.30	rho = 0.30	rho = 0.38	rho = 0.38						
	t = 1.43/NS	t = 0.42/NS	t = 4.14/***	t = 4.37/***						
Chlorophyll	rho = −0.25	rho = 0.91	rho = −0.30	rho = −0.50	rho = 0.53	rho = 0.91		rho = −0.30		rho = −0.50
	t = −1.17/NS	t = 13.81/NS	t = −3.77/NS	t = −6.50/NS	t = 1.51/NS	t = 1.38/***		t = −3.77/NS		t = −6.50/NS
CG_fish_	rho = 0.62	rho = 0.53	rho = 0.60	rho = 0.61	rho = 0.77	rho = 0.42		rho = −0.46		rho = −0.51
	t = 5.00/***	t = 4.56/***	t = 10.76/***	t = 9.35/***	t = 4.70/***	t = 1.56/NS		t = −1.85/*		t = 5.68/***

Asterisks indicate significant difference: *: <0.05; **: <0.01; ***:<0.001; NS: not significant.

The interaction between CG_macro_ and stratification at night differed between the inshore and offshore regions; the correlation was positive inshore, and negative offshore (Figs. S1 and S2, Table 1). In addition, CG_macro_ was positively correlated with CG_fish_ in the inshore region and with chlorophyll-a concentration in the offshore region (Figs. S1 and S2). Typical of the inshore region of the French area, there was a negative relationship between CG_macro_ and stratification, whereas no relationship was found in the offshore region. Once more, CG_macro_ was positively correlated with CG_fish_ in both regions (Figs. S1 and S2, Table 1).

The biocline was positively correlated with the photic depth in the inshore region of the Spanish area, and in both regions of the French area (in a similar way to CG_macro_) (Fig. S3 and Table 2). The biocline was, however, negatively correlated with the stratification index in both regions of the French area.

**Table 2:** Correlations between biocline and the environmental variables according to the diel period, the area (Spain or France) and the ecological domains (inshore, offshore).

		Biocline			
		Day			
		Spain		France	
		Inshore	Offshore	Inshore	Offshore
		(n = 25)	(n = 40)	(n = 263)	(n = 218)
Stratification		rho = 0.30	rho = 0.19	rho = −0.45	rho = −0.35
		t = 1.35/NS	t = 1.06/NS	t = −5.05/***	t = −2.49/***
Photic depth	rho = 0.47		rho = 0.070	rho = 0.42	rho = 0.60
	t = 2.05/*		t = −0.35/NS	t = 5.12/***	t = 6.75/***
Chlorophyll		rho = −0.42	rho = 0.06	rho = −0.20	rho = −0.37
		t = −1.89/NS	t = 0.30/NS	t = −2.34/NS	t = −4.40/NS
CG_fish_		rho = −0.38	rho = 0.06	rho = −0.01	rho = −0.02
		t = −2.11/NS	t = 0.41/NS	t = −0.18/NS	t = −0.32/NS

Asterisks indicate significant difference: *: <0.05; **: <0.01; ***:<0.001; NS: not significant.

**Figure 8:**
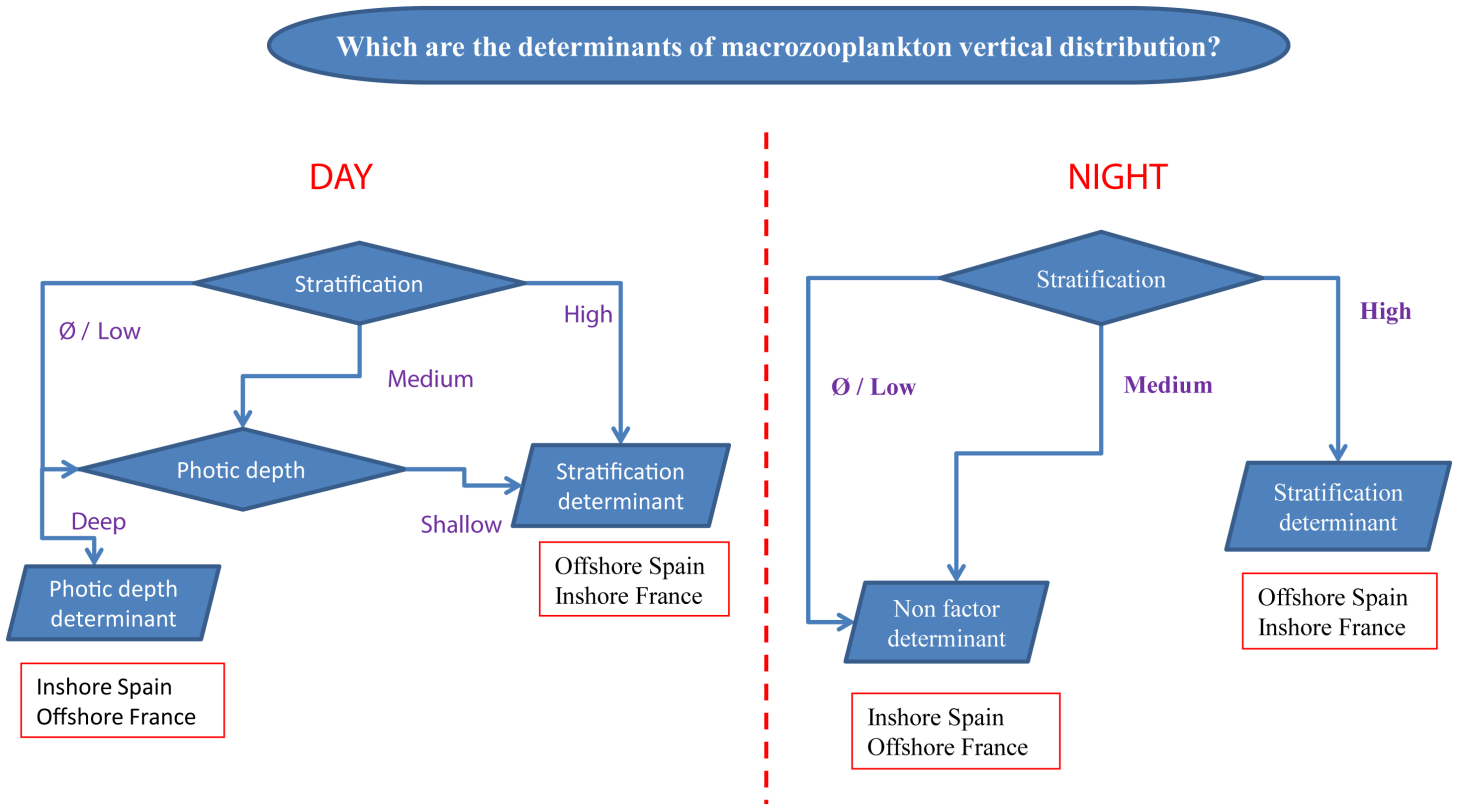
Flow chart showing the nested and interacting nature of the environmental effects on macrozooplankton.

Overall, observations at a local scale suggest that the vertical distribution patterns exhibited by macrozooplankton are consistent with that of a classic diel cycle, with deeper CG_macro_ during the day than at night regardless of the region. Furthermore, for a given diel period (day or night), it appears as though the macrozooplankton vertical distribution depends on several factors including the thermocline and halocline depth and/or strength, and the stratification and photic depth. The influence of each of these parameters, however, depends on the progress and timing of the annual spring stratification and other characteristics of the environment. We constructed a flow chart summarizing the nested and interacting nature of the environmental effects we observed during the day and night periods (Fig. 8). When stratification is high, such as was found in the inshore region of the French area or Spanish offshore region, it determines the macrozooplankton vertical distribution patterns during both diel periods. As the stratification increase, the photic depth decrease and therefore macrozooplankton tend to concentrate in shallower waters. When the stratification is weaker, associated with a less pronounced thermohaline vertical structure (e.g. French offshore region or Spanish inshore area), other factors besides stratification play an increasingly important role, such as photic depth during daytime. As the stratification decrease, the photic depth increase and therefore macrozooplankton tend to concentrate in deeper waters. However, at night and when stratification was weak, none of the environmental parameters we considered could adequately explain the vertical distribution patterns of macrozooplankton.

## Discussion

The distribution of macrozooplankton in the Bay of Biscay has been poorly documented since most of the studies carried out in the region have been based on zooplankton samples collected almost exclusively with ≤250 µm mesh size nets. Although some previous studies make reference to macrozooplankton, their main focus has actually been on the mesozoooplankton component ([47]–[51], Table 3). This study, however, which is based on acoustic data, partly removes some of the limitations pertaining to net sampling.

**Table 3:** Review of dominant species of macrozooplakton from Bay of Biscay during spring season (from literature).

Specie	Biomass & Abundance (relative)	Reference and comments
*Meganictyphanes norvegica*		[48]–[51]
*Nyctiphanes couchii*	0.22% tot. zoopk abundance	
*Thysanoessa longicaudata*		
*Calanus helgolandicus*	0.58 and 0.36% tot. zoopk abundance	[48]–[51]
*Calanoides carinatus*	40.4% tot. copepod biomass	
Candacia sp.	0.06% of tot. zooplankton abundance	[48]–[51]

All studies encompass day and night data and a sampled depth range of 100 m (method: 150- µm PairoVET net).

The most important finding of this study is, the fact that through the use of acoustic data, the presence of a ‘biocline’ during the day was discovered. It is defined as the interface separating the surface layer, almost deplete of macrozooplankton, from the macrozooplankton-rich deeper layer. This study is further focussed on the vertical behaviour of the macrozooplankton community (i.e., zooplankton >∼2 mm), composed mainly of big and conspicuous individuals such as large copepods (*Calanus spp.*) and euphausiids (*Meganictyphanes norvegica*) (Table 3), and which play an important ecological role in the total biomass of zooplankton during the spring season [47]–[51].

The biocline observed during the day can be considered a specific feature that fits in with the ‘classic’ pattern of diel vertical migration. Indeed vertical migration was clearly evident with the bulk of the macrozooplankton distributed in the deeper depth strata during the day (Fig. 3). A similar study off Peru [17] observed that 79% of the macrozooplankton migrated vertically, but that the surface layer was always occupied by non-migrant organisms during the day. The biocline, observed in the Bay of Biscay, which was associated with a surface layer devoid of macrozooplankton, is therefore associated with the vertical structuring of the ecosystem during the diel vertical migration.

Diel vertical migration is generally thought to minimize spatiotemporal overlap with visually hunting predators in surface strata during daylight hours. The risk of attacks by planktivorous fish increases with ambient light level, but also depends on characteristics of the prey that affect visibility such as body size, morphology, pigmentation, mobility patterns, and gut contents [10], [13], [52]–[56]. Thus, large-bodied and highly pigmented organisms such as macrozooplankton are extremely vulnerable to visual predators [13], [52], [56]. In this context, the macrozooplankton biocline could potentially be seen as that position in the water column that optimises the trade off between avoiding size-selective visually hunting predators and maximizing energy gain.

The biocline depth generally coincided with the thermocline depth, associated with the strongest temperature vertical gradients, except over the slope. Once the stratification process was enhanced, and relatively strong stratification levels were reached in the thermocline, the biocline coincided with the depth of maximum stratification. This suggested that the thermohaline vertical structure and stratification process can strongly impact the spatial distribution patterns of plankton communities [57]. During the day, the distribution of macrozooplankton below the thermocline suggests that once the risk of visual predation is reduced by moving to deeper darker layers, there is an apparent metabolic benefit for the macrozooplankton of staying in the colder waters below the thermocline.

Both the biocline and the depth of the bulk of the macrozooplankton (CG_macro_) deepened over time, coinciding with spatiotemporal variations in the depth of the thermocline and halocline. In addition, the deepening of the macrozooplankton distribution toward the offshore parts of the study area was linked to the rather weak and shallow thermocline in these regions and the lack of a marked halocline.

Chlorophyll-a concentration had only a local effect on macrozooplankton patterns. An increase in chlorophyll-a concentration associated with river plumes (the signal may have been caused by turbidity and yellow substances instead of Chlorophyll-a) led to a deeper distribution of macrozooplankton, probably brought about by migratory behaviour in an attempt to avoid the relatively fresh surface water. At the shelf break, where a deep chlorophyll-a maximum occurs [58], the biocline and CG_macro_ were also distributed deeper, possibly suggesting that macrozooplankton prefer to use this resource rather than migrating all the way to the surface [12].

Although day/night changes were indeed the dominant factor affecting the vertical distribution, other factors were important in explaining the observed patterns. Photic depth was a determinant factor for explaining the biocline and CG_macro_ distributions when stratification was weak, i.e., in the Spanish inshore region and French offshore region. In cases where stratification was well established or there was a local increase in dissolved particles (blooms, river discharges, etc), this parameter had no or little effect on the vertical distribution of macrozooplankton. A similar observation has previously been noted [59]–[61]. Due to river run-off and the influx of particles and dissolved organic substances in the coastal area, the penetration of light into the water column is much lower than at the shelf-break (typically reduced 10-fold; Guillem Chust, personal communication). This may impact predator-prey relationships. It may, however, also be a shortcoming of this study since in stratified waters, associated with a near-surface layer rich in chlorophyll-a, satellite estimates of light attenuation below this layer are unreliable [62].

The vertical distribution of fish was the only factor that could act as a proxy for both a cause and/or a response to macrozooplankton distribution. The distribution of macrozooplankton throughout the water column was more homogenous (higher inertia around the centre of gravity) in offshore regions compared to the coastal regions for both diel periods. This may be a response to fish absence, as observed in the offshore regions, since macrozooplankton tend to have a more homogeneous distribution throughout the water column when there is no need for swarming, because predation pressure is low [63]. In general though, the vertical distribution of fish coincided with that of the macrozooplankton, which in turn appeared to be influenced by the vertical physical structure. This suggests that fish track their prey movements, but obviously their predatory efficiency changes with light level.

### 

#### Summary

Information on macrozooplankton is scarce, particularly at high-resolution, which prevents a full understanding of its distribution and ecological role in the Bay of Biscay ecosystem. The continuous, high resolution information provided by the acoustic method allowed us to define the biocline as the upper limit of the macroozooplankton vertical distribution and investigate its relationship with different environmental parameters and predation. This study used data from only one survey, though and more data are necessary to fully understand the processes responsible for macrozooplankton distribution. Our observations do, however, suggest the following: (i) the depth of the biocline, which was only present during the day, was related to the depth and structure of the thermocline (except in the slope region); (ii) when stratification was intense, the biocline depth was closely associated with the depth of maximum stratification; and (iii) the biocline depth coincided with the photic depth in regions where light transmission in the water column increased. Furthermore, the vertical overlap between fish backscattering and biocline indicated that the vertical distribution of fish in the Bay of Biscay tracks that of the macrozooplankton distribution. The high presence of fish and the lack of food in the surface layer, force macrozooplankton to deeper colder waters where their metabolic demand is lower and the risk of predation is reduced. The biocline is therefore assumed to have developed as an adaptive response to the environmental conditions (with the exception of the slope region where deep peaks of phytoplankton exist). The reduction of light needed to reduce visibility and counter predation may be reached at shallower depth than that of the biocline, but all organisms compensate metabolically by inhabiting colder water. Although the biocline has not been previously described, it is possible that such a vertical structure also occurs in systems other than the Bay of Biscay.

## Supporting Information

Figure S1 **Scatter plots of the correlations between CG_macro_ and environmental variables (stratification, photic depth and CG_fish_), in relation to areas (Spanish and French) and ecological domains (inshore-offshore) during the day period.** Only significant relationships are presented. Scatter plots include a linear fit (black solid line) to illustrate the sign of the correlation.
(TIF)

Figure S2 **Scatter plots of the correlations between CG_macro_ and environmental variables (stratification, chlorophyll-a, and CG_fish_), in relation to areas (Spanish and French) and ecological domains (inshore-offshore) during the night period.** Only significant relationships are presented. Scatter plots include a linear fit (black solid line) to illustrate the sign of the correlation.
(TIF)

Figure S3 **Scatter plots of the correlations between biocline and environmental variables (stratification and photic depth), in relation to areas (Spanish and French) and ecological domains (inshore-offshore) during the night period.** Only significant relationships are presented. Scatter plots include a linear fit (black solid line) to illustrate the sign of the correlation.
(TIF)
