# Supplementary figures and images for: Acoustics Reveals the Presence of a Macrozooplankton Biocline in the Bay of Biscay in Response to Hydrological Conditions and Predator-Prey Relationships
Source: PLoS One. 2014 Feb 4;9(2):e88054. doi: 10.1371/journal.pone.0088054 (PMC3913746; doi:10.1371/journal.pone.0088054)

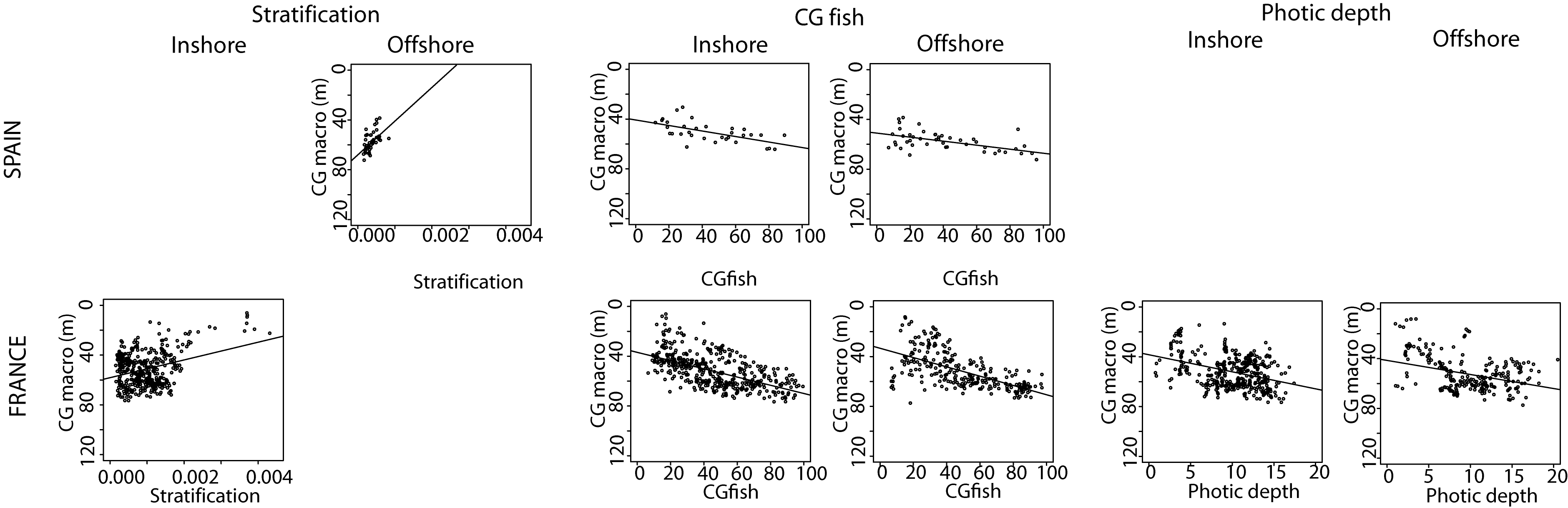

Supplement: Figure S1 — Scatter plots of the correlations between CGmacro and environmental variables (stratification, photic depth and CGfish), in relation to areas (Spanish and French) and ecological domains (inshore-offshore) during the day period. Only significant relationships are presented. Scatter plots include a linear fit (black solid line) to illustrate the sign of the correlation. (TIF) [file pone.0088054.s001.tif]

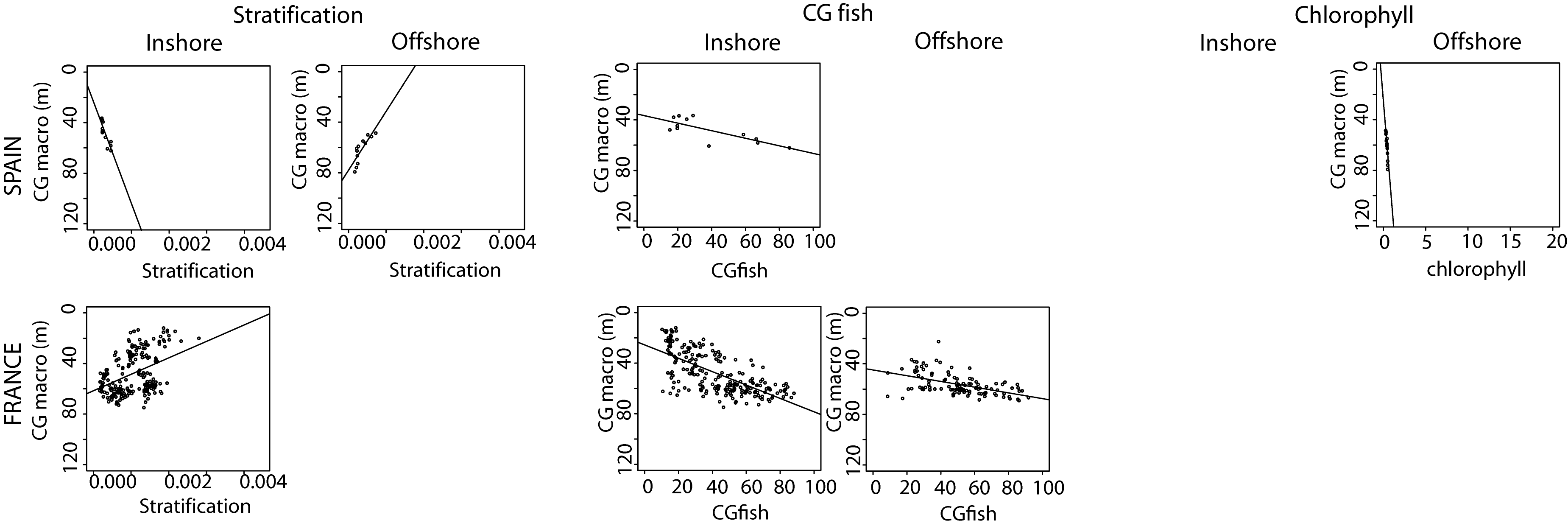

Supplement: Figure S2 — Scatter plots of the correlations between CGmacro and environmental variables (stratification, chlorophyll-a, and CGfish), in relation to areas (Spanish and French) and ecological domains (inshore-offshore) during the night period. Only significant relationships are presented. Scatter plots include a linear fit (black solid line) to illustrate the sign of the correlation. (TIF) [file pone.0088054.s002.tif]

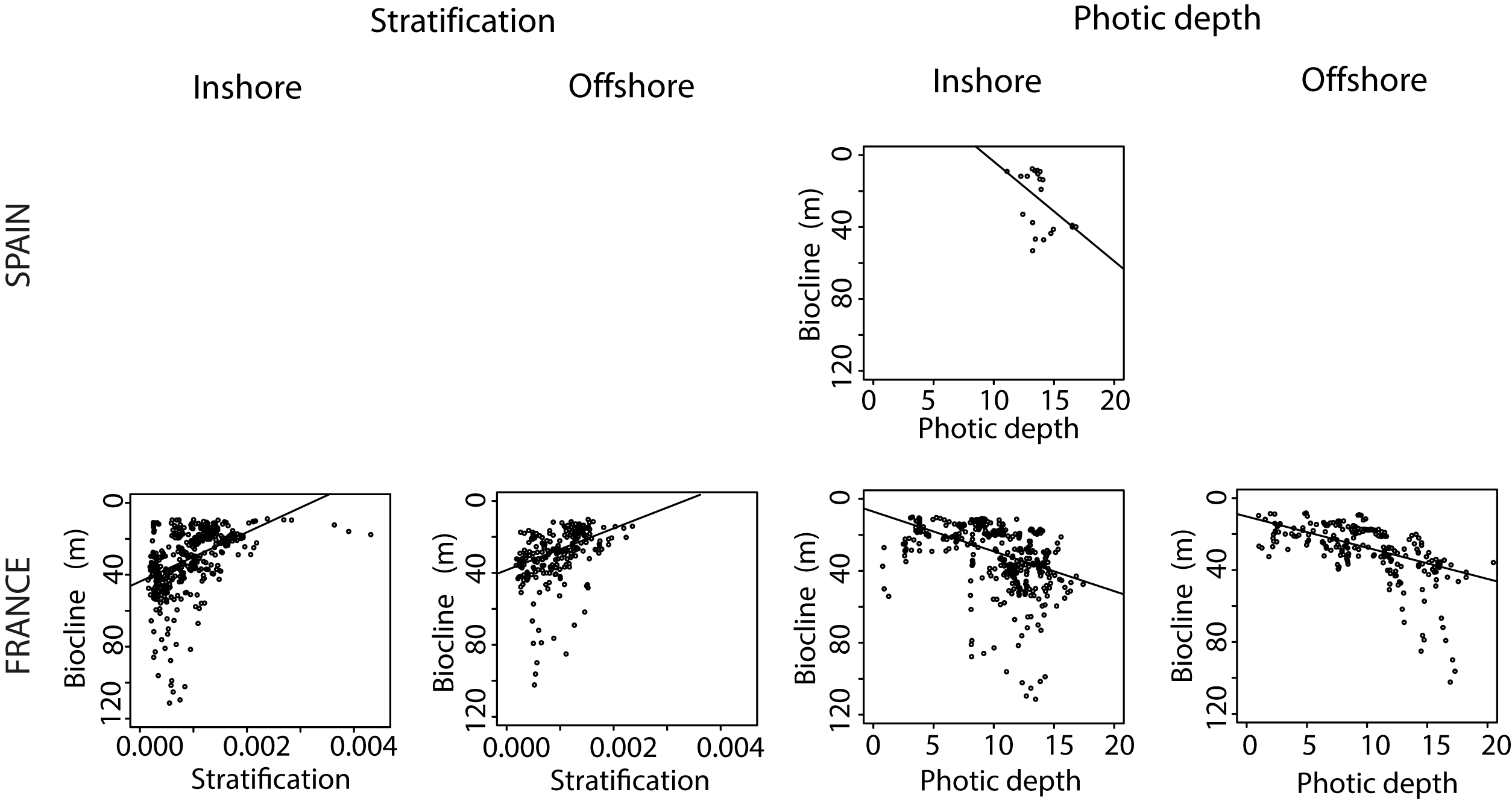

Supplement: Figure S3 — Scatter plots of the correlations between biocline and environmental variables (stratification and photic depth), in relation to areas (Spanish and French) and ecological domains (inshore-offshore) during the night period. Only significant relationships are presented. Scatter plots include a linear fit (black solid line) to illustrate the sign of the correlation. (TIF) [file pone.0088054.s003.tif]
